# Supplementary material for: Centriole triplet microtubules are required for stable centriole formation and inheritance in human cells
Source: eLife. 2017 Sep 14;6:e29061. doi: 10.7554/eLife.29061 (PMC5653238; doi:10.7554/eLife.29061)
Supplement: Figure 4—source data 1. — Accession numbers and references for evolutionary analyses. [file elife-29061-fig4-data1.docx]

**Figure 4 – source data 1: Expanded evolutionary analysis**

| **Genus** | **TUBD1 accession** | **TUBE1 accession** | **Centriolar microtubule reference** |
| --- | --- | --- | --- |
| Trichonympha | AB819960 | AB819961 | (Guichard et al., 2013) |
| Rozella | EPZ31138 | EPZ34661 | (Held, 1975) |
| Caenorhabditis | None | None | (Nechipurenko et al., 2017; Serwas et al., 2017) |
| Apis | XP_394700 | XP_003250659 | (Hoage and Kessel, 1968) |
| Drosophila | None | None | (Gottardo et al., 2015) |
| Homo | NP_057346 | NP_057345 | (Vorobjev and Chentsov, 1980; Paintrand et al., 1992) |
| Trypanosoma | XP_822372 | XP_829157 | (McKean et al., 2003) |
| Chlamydomonas | XP_001703303 | AAB71840 | (Ringo, 1967; Li et al., 2012) |
| Physcomitrella | XP_001784101 | XP_001753613 | (Doonan et al., 1986) |
| Ginkgo | None | None | (Gifford and Lin, 1975) |
| Paramecium | XP_001437029 | XP_001429943 | (Dippell, 1968) |
| Tetrahymena | XP_001010767 | XP_001017563 | (Allen, 1969) |

**References**

Allen RD. 1969. The morphogenesis of basal bodies and accessory structures of the cortex of the ciliated protozoan Tetrahymena pyriformis. *The Journal of Cell Biology* **40**:716–733. DOI: <https://doi.org/10.1083/jcb.40.3.716>, PMID: 5765762

Dippell RV. 1968. The development of basal bodies in paramecium. *PNAS* **61**:461–468. DOI: <https://doi.org/10.1073/pnas.61.2.461>, PMID: 4176480

Doonan JH, Lloyd CW, Duckett JG. 1986. Anti-tubulin antibodies locate the blepharoplast during spermatogenesis in the fern Platyzoma microphyllum R.Br.: a correlated immunofluorescence and electron-microscopic study. *Journal of Cell Science* **81**:243–265. PMID: 3525583

Gifford EM, Lin J. 1975. Light microscope and ultrastructural studies of the male gametophyte in ginkgo biloba: The spermatogenous cell. *American Journal of Botany* **62**:974. DOI: <https://doi.org/10.2307/2441642>

Gottardo M, Callaini G, Riparbelli MG. 2015. The Drosophila centriole - conversion of doublets into triplets within the stem cell niche. *Journal of Cell Science* **128**:2437–2442. DOI: <https://doi.org/10.1242/jcs.172627>, PMID: 26092937

Guichard P, Hachet V, Majubu N, Neves A, Demurtas D, Olieric N, Fluckiger I, Yamada A, Kihara K, Nishida Y, Moriya S, Steinmetz MO, Hongoh Y, Gönczy P. 2013. Native architecture of the centriole proximal region reveals features underlying its 9-fold radial symmetry. *Current Biology* 23:1620–1628. DOI: <https://doi.org/10.1016/j.cub.2013.06.061>, PMID: 23932403

Held AA. 1975. The zoospore of Rozella allomycis : ultrastructure. *Canadian Journal of Botany* **53**:2212–2232. DOI: <https://doi.org/10.1139/b75-245>

Hoage TR, Kessel RG. 1968. An electron microscope study of the process of differentiation during spermatogenesis in the drone honey bee (Apis mellifera L.) with special reference to centriole replication and elimination. *Journal of Ultrastructure Research* **24**:6–32. DOI: <https://doi.org/10.1016/S0022-5320(68)80014-0>, PMID: 5683704

Li S, Fernandez J-J, Marshall WF, Agard DA. 2012. Three-dimensional structure of basal body triplet revealed by electron cryo-tomography. *The EMBO Journal* **31**:552–562. DOI: <https://doi.org/10.1038/emboj.2011.460>

McKean PG, Baines A, Vaughan S, Gull K. 2003. Gamma-tubulin functions in the nucleation of a discrete subset of microtubules in the eukaryotic flagellum. *Current Biology* **13**:598–602. DOI: <https://doi.org/10.1016/S0960-9822(03)00174-X>, PMID: 12676092

Nechipurenko IV, Berciu C, Sengupta P, Nicastro D. 2017. Centriolar remodeling underlies basal body maturation during ciliogenesis in Caenorhabditis elegans. *eLife* **6**:e25686. DOI: <https://doi.org/10.7554/eLife.25686>, PMID: 28411364

Paintrand M, Moudjou M, Delacroix H, Bornens M. 1992. Centrosome organization and centriole architecture: their sensitivity to divalent cations. *Journal of Structural Biology* **108**:107–128. DOI: <https://doi.org/10.1016/1047-8477(92)90011-X>, PMID: 1486002

Ringo DL. 1967. Flagellar motion and fine structure of the flagellar apparatus in Chlamydomonas. *The Journal of Cell Biology* **33**:543–571. DOI: <https://doi.org/10.1083/jcb.33.3.543>, PMID: 5341020

Serwas D, Su TY, Roessler M, Wang S, Dammermann A. 2017. Centrioles initiate cilia assembly but are dispensable for maturation and maintenance in C. elegans. *The Journal of Cell Biology* **216**:1659–1671. DOI: <https://doi.org/10.1083/jcb.201610070>, PMID: 28411189

Vorobjev IA, Chentsov YS. 1980. The ultrastructure of centriole in mammalian tissue culture cells. *Cell Biology International Reports* **4**:1037–1044. DOI: <https://doi.org/10.1016/0309-651(80)90177-0>, PMID: 7438223
